# The microbial RNA metagenome of *Aedes albopictus* (Diptera: Culicidae) from Germany

**DOI:** 10.1007/s00436-022-07576-7

**Published:** 2022-07-20

**Authors:** Janine Rau, Doreen Werner, Martin Beer, Dirk Höper, Helge Kampen

**Affiliations:** 1 Friedrich-Loeffler-Institut, Federal Research Institute for Animal Health, Greifswald, Germany; 2 Leibniz Centre for Agricultural Landscape Research, Muencheberg, Germany

**Keywords:** *Aedes albopictus*, Microbiome, Germany, Vector control

## Abstract

*Aedes albopictus* is a highly invasive mosquito species that has become widespread across the globe. In addition, it is an efficient vector of numerous pathogens of medical and veterinary importance, including dengue, chikungunya and Zika viruses. Among others, the vector potential of mosquitoes is influenced by their microbiome. However, this influence is very dynamic and can vary between individuals and life stages. To obtain a rough overview on the microbiome of *Ae. albopictus* populations in Germany, pooled female and pooled male individuals from seven German locations were investigated by total RNA sequencing. The mosquito specimens had been collected as larvae in the field and processed immediately after adult emergence, i.e. without females having fed on blood. RNA fragments with high degrees of identity to a large number of viruses and microorganisms were identified, including, for example, *Wolbachia pipientis* and *Acinetobacter baumannii*, with differences between male and female mosquitoes. Knowledge about the natural occurrence of microorganisms in mosquitoes may be translated into new approaches to vector control, for example *W. pipientis* can be exploited to manipulate mosquito reproduction and vector competence. The study results show how diverse the microbiome of *Ae. albopictus* can be, and the more so needs to be adequately analysed and interpreted.

**Supplementary Information:**

The online version contains supplementary material available at 10.1007/s00436-022-07576-7.

## Introduction

*Aedes albopictus* is a thermophilic mosquito species native to the Asian-Pacific region. Due to globalisation and its high ecological and physiological plasticity, it has become widespread in many regions in the world. Presently, *Ae. albopictus* is considered the most invasive mosquito species of the world (Benedict et al. 2007; Bonizzoni et al. 2013). Climate warming and the resulting mild winters favour the establishment, reproduction and spread of *Ae. albopictus* in temperate climes, such as Central Europe (e.g., Walther et al. 2017; Fălcuţă et al. 2020).

*Aedes albopictus* is highly vector-competent for numerous arboviruses, including dengue, chikungunya, yellow fever, Zika, West Nile and various encephalitis viruses (Paupy et al. 2009; Martinet et al. 2019). It thus has a major impact on human and veterinary health. The vector competence, i.e. the ability of a haematophagous arthropod to transmit a pathogen, can be influenced by the arthropod’s microbiome (Engel and Moran 2013; Jupatanakul et al. 2014) which is defined by Berg et al. (2020) as ‘a characteristic microbial community occupying a reasonable well-defined habitat which has distinct physio-chemical properties’.

It has been shown that the microbiome may have a general impact on the development, reproduction and physiology of an invertebrate (Minard et al. 2013; Coon et al. 2014, 2016, 2017). For example, the endosymbiont *Wolbachia pipientis* is known to be widely distributed in invertebrates (Yang 2000; Hilgenboecker et al. 2008). Bourtzis and O’Neill (1998) and Ahmad et al. (2017) have demonstrated that *W. pipientis* can affect both the reproduction of insects and the replication and dissemination of pathogenic viruses in an insect vector. These effects are major reasons why the study of the microbiome of mosquitoes has become so popular in recent years. However, the microbiome is not static, but may change during development and can be influenced by many factors such as sex, age and life stage of the host, geographic location, breeding habitat characteristics and food supply (Wang et al. 2011; Zouache et al. 2011; Boissière et al. 2012; Terenius et al. 2012; Jupatanakul et al. 2014; Chen et al. 2020). Water temperature and nutrient content of the breeding habitat, for example, can strongly influence its bacterial community and thus have an impact on the microbiome of developing mosquito larvae (Hörtnagl et al. 2010; Onchuru et al. 2016). In turn, the microbiome ingested from the breeding habitat may considerably influence larval growth and development (Coon et al. 2014, 2016, 2017).

Insects take up a variety of microorganisms from their environment (Strand 2018). In the case of mosquitoes, this mainly occurs in the larval stage, when individuals are confronted with large numbers of microorganisms in their aquatic habitats during feeding. Larval nutrition can therefore have a major impact on the composition of the microbiome (Wang et al. 2011; Boissiere et al. 2012; Coon et al. 2016). By contrast, occasions to take up microorganisms in the adult stage are limited: both sexes feed on sugary plant juices and only females feed on blood, with the latter occasionally facilitating the uptake of disease agents. There is evidence that the insect host can exert some control over its microbiome via the innate immune response (Douglas 2015; Smith et al. 2015).

In recent years, the microbiome of several mosquito species has been studied, among them *Ae. albopictus*, *Aedes japonicus*, *Anopheles gambiae* and *Culex pipiens*, with the focus of most studies being on the midgut microbiota of adult mosquitoes (Wang et al. 2011; Gimonneau et al. 2014). It turned out that the microbiome of some species is extremely diverse, and a variety of bacterial phyla such as Actinobacteria, Proteobacteria, Bacteroidetes or Firmicutes could be detected (Moro et al 2013; Zotzmann et al. 2017; Wang et al. 2018).

The extent to which the microbiome of a species differs between populations and individuals is largely unexplored. Furthermore, nothing is known about microorganisms naturally occurring in *Ae. albopictus* in Germany, the influence they have on their host and whether they pose a threat to humans or may be exploited to their benefit. This study presents the first preliminary insights into the microbial RNA metagenome of *Ae. albopictus* from Germany which can be considered to represent the mosquito’s microbiome. When interpreting the results, however, it is essential to keep in mind that RNA reads similar to a certain microbial species vary considerably in number, and contigs generated from them are of various lengths and have various degrees of probability to be identical to a certain microbial species.

## Materials and methods

### Mosquito origin

**Fig. 1:**
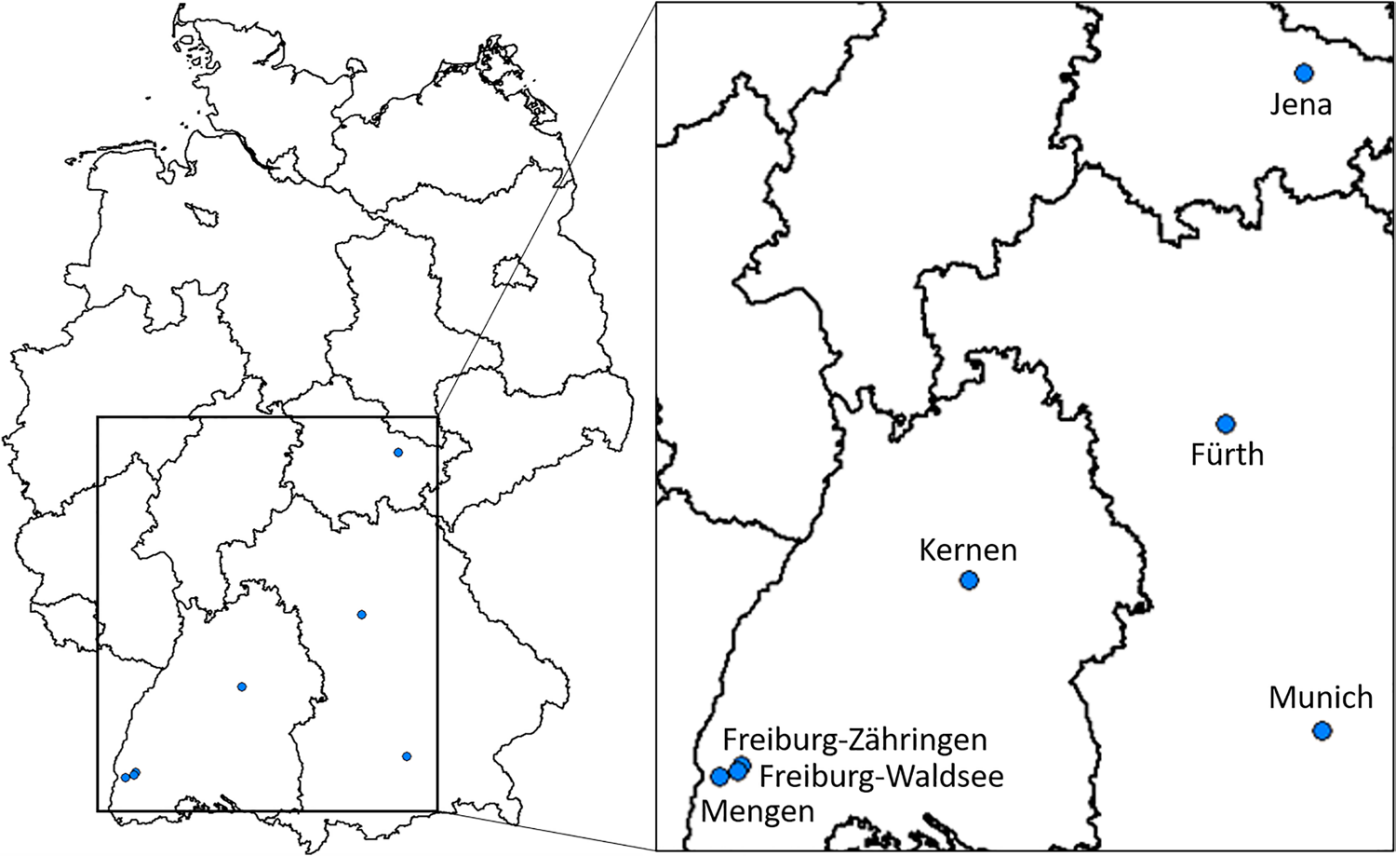
Collection sites in Germany of *Ae. albopictus* specimens examined (blue dots)

The *Ae. albopictus* specimens investigated in this study had been collected as larvae in the field at seven sites in Germany in 2020 (Fig. 1): Mengen, Freiburg-Waldsee, Freiburg-Zähringen, Kernen, Munich, Fürth and Jena. These were the locations successfully checked for the presence of *Ae. albopictus* aquatic stages from all German cities known to possess established populations at the time of the study. Individuals were obtained by sieving potential breeding containers in cemeteries (Mengen, Freiburg-Waldsee, Freiburg-Zähringen, Munich, Jena), in gardens of a settlement (Kernen) and an allotment garden complex (Fürth).

Collected larvae were kept in water from their breeding habitat until adult emergence while being fed ad libitum with ground TabiMin fish food pellets (Tetra, Melle, Germany). Shortly after hatching, adults were killed by freezing at − 20 °C, without being offered blood or a sugar solution. They were morphologically identified on a chilling table using a stereomicroscope according to the determination key by Becker et al. (2010) and stored in 75% ethanol until further processing. Except for the Freiburg-Zähringen site, from where only one female and no male were available, two females and two males per site were analysed.

### Nucleic acid extraction and sequencing

For nucleic acid extraction, the mosquitoes were removed from the ethanol and dried for about 1 min at room temperature for the alcohol to evaporate. Subsequently, 13 female and 12 male mosquitoes were pooled by sex and then completely homogenised in 500 µl serum-free ZB5d medium (FLI-intern cell culture medium = Eagle’s minimal essential medium with Earle’s and Hank’s salts plus non-essential amino acids) containing 5 µl of a ready-to-use mixture of penicillin–streptomycin and 1 µl of a ready-to-use mixture of gentamicin-amphotericin (Thermo Fisher Scientific, Dreieich, Germany). Three steel beads with a diameter of 3 mm (TIS GmbH, Gauting, Germany) were added and the samples agitated for 2 min at 30 Hz in a TissueLyser II (Qiagen, Hilden, Germany). Nucleic acid was then extracted from 200 µl of the supernatant using the NucleoMag VET kit (Macherey–Nagel, Düren, Germany) according to the manufacturer’s instructions, but without the addition of carrier RNA. The concentration of extracted RNA (12.2 ng/µl for the female sample, 11.3 ng/µl for the male sample) was measured using a NanoDrop Lite (Thermo Fisher Scientific).

Further processing of the sample for total RNA sequencing with Ion Torrent technology, including manual library preparation, was performed following the protocol described by Wylezich et al. (2018). Briefly, the extracted RNA was transcribed into double-stranded cDNA using the cDNA Synthesis System Kit (Roche, Mannheim, Germany), then fragmented by an M220 Focused-ultrasonicator (Covaris Ltd., Brighton, UK) and prepared for Ion Torrent-compatible library generation by means of the GeneRead L Core Kit (Qiagen) and Ion Xpress barcode adapters (Thermo Fisher Scientific). The resulting library was subjected to quality control in a 2100 Bioanalyzer (Agilent Technologies, Santa Clara, USA), using the High Sensitivity DNA Kit (Agilent Technologies) and quantification with a KAPA Library Quantification Kit (Roche). The library was then sequenced on an Ion Torrent S5 XL System (Thermo Fisher Scientific) according to the manufacturer’s instructions.

### Data analysis

Sequencing results were edited and analysed using Geneious Prime version 2021.0.1 (Biomatters, Auckland, New Zealand). For this, sequences trimmed to a minimum length of 25 bp by the Geneious BBDuk tool were merged by the BBMerge tool, using a merge rate set to ‘high’. Both merged data and data that could not be merged were assembled de novo to cluster all closely related sequences into contigs, based on a ‘custom sensitivity’ setting. The obtained consensus sequences were sorted according to their length, resulting in groups of contigs with similar base pair length. Each contig of these groups was subsequently aligned with GenBank entries (www.ncbi.nlm.nih.gov), with equal results being summarised.

## Results

**Fig. 2:**
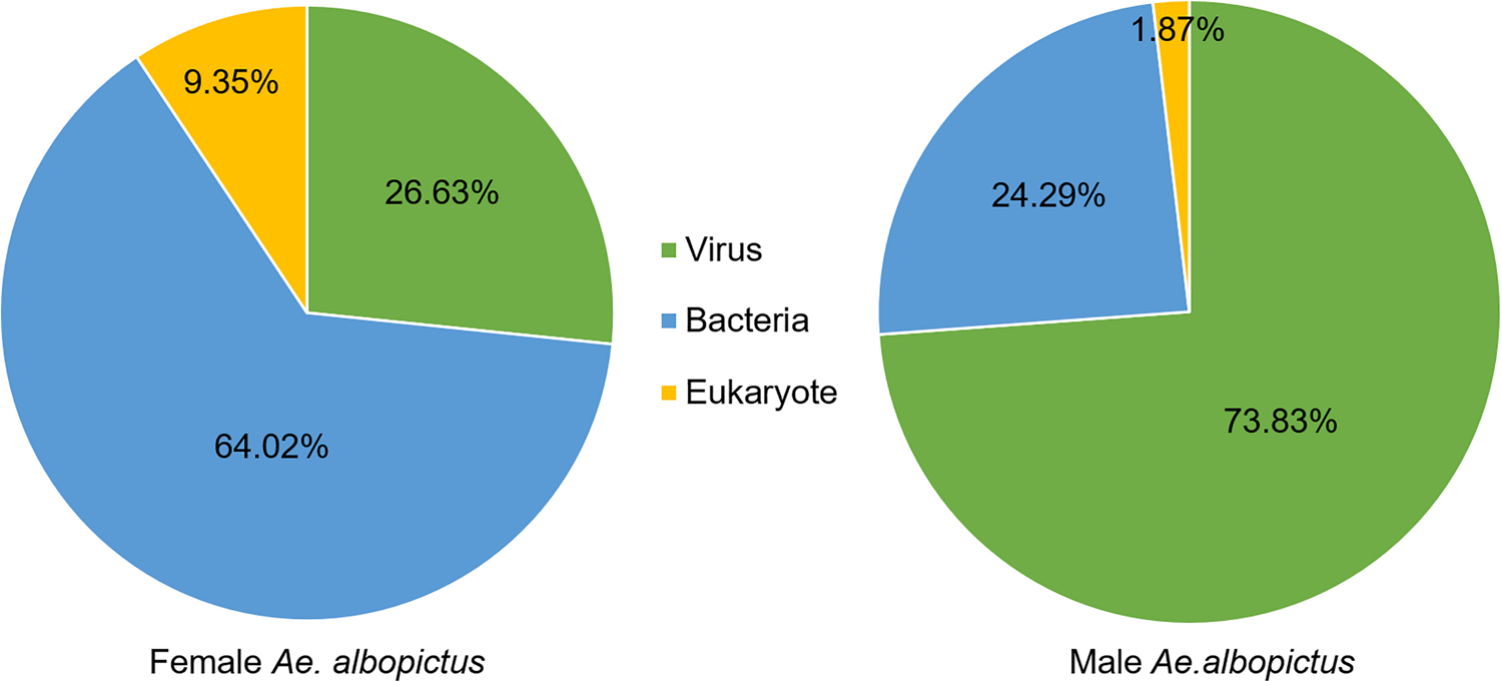
Percentage of contigs from RNA reads from *Ae. albopictus* females and males according to viral, bacterial and eukaryotic origin

A total of 5,000,504 RNA reads were generated for the female *Ae. albopictus* pool and 4,653,856 reads for the male pool. In the microbial RNA metagenome of female mosquitoes, RNA fragments with high identities to 42 different microorganismal species from 37 different families were detected, whereas in males RNAs with high identities to a total of 38 different species from 36 families were found (Table 1, Supplementary Tables 1 and 2). In the pool of female mosquitoes, there were a total of 213 contigs (in the range of 69.91–100% percent identity (p.i.), 91–100% query cover and 32–1674 bp), 20 (9.35%) of which matched with eukaryotic, 136 (64.02%) with bacterial and 57 (26.63%) with viral species. In the male mosquito pool, a total of 1380 contigs (82.99–100% p.i., 96–100% query cover, 28—1702 bp) was analysed, 19 (1.87%) of which could be assigned to eukaryotes, 338 (24.29%) to bacteria and 1023 (73.83%) to viruses (Fig. 2). Often, an identification at the species level was not possible and only the genus could be determined.

**Table 1:** Species of viruses/microorganisms whose RNA matched that isolated from *Ae. albopictus* to at least 97% (see Supplementary Tables 1 and 2 for more details and species with RNA identity lower than 97%)

Species	Phylum	Found in sex	Remark
**Viruses**			
Aedes albopictus anphevirus	Riboviria	♂♀	Insect-specific virus; previously detected in *Ae. albopictus* (Manni and Zdobnov 2020)
Aedes phasmavirus	Riboviria	♂♀	Previously detected in *Ae. albopictus* (Shi et al. 2020)
Barstukas virus	Riboviria	♂♀	Previously detected in various *Aedes* mosquitoes (Batson et al. 2021)
GuapiaÇu virus	Riboviria	♂♀	Insect-specific virus; previously detected in *Ae. terrens* and *Ae. scapularis* (Batson et al. 2021; Oliveira Ribeiro et al. 2021)
High Island virus	Riboviria	♂♀	Previously detected in mosquitoes and other invertebrates (Sadeghi et al. 2017)
Usinis virus	Riboviria	♂♀	Previously detected in *Ae. albopictus* (Batson et al. 2021)
Wenzhou sobemo-like virus	Riboviria	♂♀	Previously detected in *Ae. albopictus* (Kubacki et al. 2020)
**Bacteria**			
*Acidovorax avenea*	Proteobacteria	♀	Plant pathogen (Walcott and Gitaitis 2000)
*Acinetobacter baumannii*	Proteobacteria	♂♀	Human pathogen; previously detected in *Ae. albopictus* (Minard et al. 2013)
*Acinetobacter dispersus*	Proteobacteria	♂	Previously detected on human skin and human wounds, in water and soil (Nemec et al. 2016)
*Acinetobacter johnsonii*	Proteobacteria	♂♀	Human pathogen; previously detected in *Ae. albopictus* and other mosquito species (Seifert et al. 1993; Minard et al. 2013)
*Acinetobacter oleivorans*	Proteobacteria	♂	Previously detected in soil (Uniyal et al. 2016)
*Acinetobacter tandoii*	Proteobacteria	♀	Previously detected in termites (van Dexter and Boopathy 2019)
*Aeromonas hydrophila*	Proteobacteria	♀	Pathogenic to many different vertebrates and humans; lives in water habitats (Emerson and Norris 1905; Wohlgemut et al. 1970; Hazen et al. 1978; Agger et al. 1985)
*Arthrobacter woluwensis*	Actinobacteria	♀	Potential human pathogen (Bernasconi et al. 2004; Li et al. 2021)
*Chryseobacterium aureum*	Bacteroidetes	♂	Previously detected in river water in Korea (Lee et al. 2019)
*Chryseobacterium indoltheticum*	Bacteroidetes	♂	Potential human pathogen; previously detected in marine mud (Calderón et al. 2011)
*Chryseobacterium scophthalmum*	Bacteroidetes	♂	Fish pathogen (Shahi et al. 2018)
*Elizabethkingia anophelis*	Bacteroidetes	♂♀	Human pathogen: previously detected in *An. gambiae* (Kämpfer et al. 2011)
*Escherichia coli*	Proteobacteria	♂♀	Intestinal bacterium; previously detected in *An. funestus* (Straif et al. 1989)
*Hydrogenophaga pseudoflava*	Proteobacteria	♀	Previously detected in the midgut of *An. gambiae* (Straif et al. 1989)
*Leclercia adecarboxylata*	Proteobacteria	♂	Potential human pathogen (Hess et al. 2008)
*Limnobacter humi*	Proteobacteria	♂	Previously detected in humus soil (Nguyen and Kim 2017)
*Micrococcus luteus*	Actinobacteria	♀	Potential human pathogen (Fosse et al. 1985)
*Paracoccus yeei*	Proteobacteria	♀	Human pathogen; previously detected in the salivary glands of *Ae. aegypti* (Arias and Clark 2019; Balaji et al. 2021)
*Pseudomonas luteola*	Proteobacteria	♀	Human pathogen; previously detected in humid environments (Kostmann et al. 1990; Altinok et al. 2007)
*Serratia marcescens*	Proteobacteria	♂	Human pathogen; previously detected in *Anopheles* mosquitoes (Hejazi and Falkiner 1997; Bai et al. 2019)
*Wolbachia pipientis*	Proteobacteria	♂♀	Previously detected in *Ae. albopictus* (Wiwatanaratanabutr 2013)
*Zooglea resiniphila*	Proteobacteria	♂	Previously detected in activated sludge (An et al. 2016)
**Eukaryota**			
*Candida sake*	Ascomycota	♂♀	Previously detected in oral cavity of HIV-positive people (Hoegl et al. 1998)
*Conidiobolus coronatus*	Zoopagomycota	♀	Potential human pathogen; previously detected on dead leaf (Fischer et al. 2008)

**Fig. 3:**
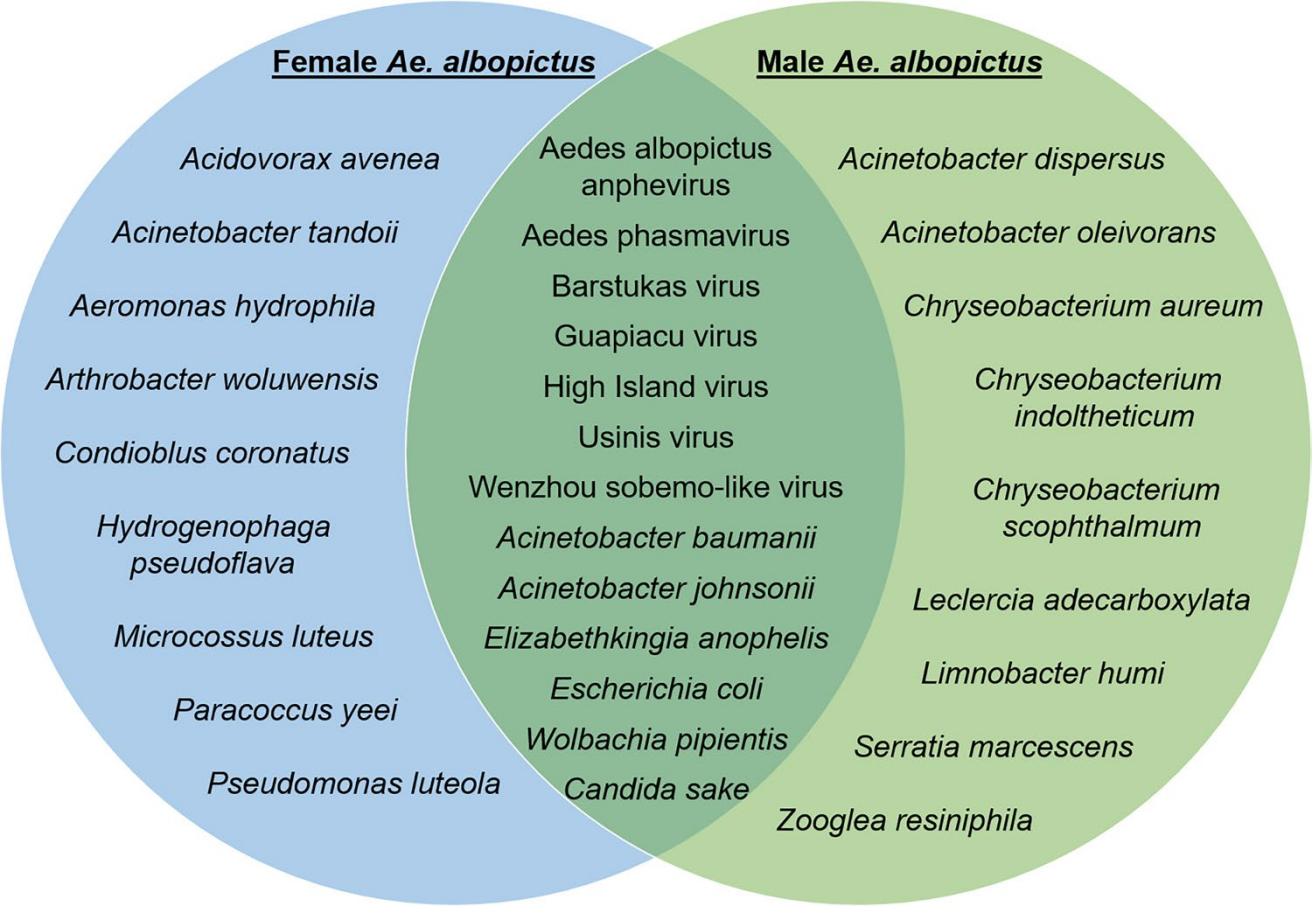
Assignment of RNA contigs obtained from *Ae. albopictus* to viral, bacterial and eukaryotic species, according to mosquito sex

Contigs with high identities to 13 species and 12 genera of viruses/microorganisms were identified in mosquitoes of both sexes (Fig. 3). Contigs arguing for seven microbial species formerly described in *Ae. albopictus* were found in both female and male mosquitoes. Four additional microbial species suggested by the contigs had been found in other mosquito species previously. Another two species of bacterial contigs female *Ae. albopictus* were suggestive of had also been found in other mosquito species, but were not present in our pool of male mosquitoes. Instead, a bacterial species indicated by contigs found in the male pool, but not in the female one of this study, had previously been detected in other mosquito species.

Table 1 shows all viruses and microorganisms whose RNA was identified in *Ae. albopictus* with a p.i. of at least 97% and a query cover of 100%, representing a minimum contig length of 56 bp. RNAs with less p.i. and query cover, shorter lengths or RNAs that could not be assigned to a species, but only to a genus or higher systematic level, are listed in Supplementary Tables 1 and 2. Only the first group of RNAs is discussed in the following.

RNAs with identity to Aedes albopictus anphevirus (89.23–100% p.i., 42–370 bp, 281 contigs) and Aedes phasmavirus (96.57–100% p.i., 30–350 bp, 13 contigs) were detected in both female and male *Ae. albopictus* (Table 1). Both viruses are considered insect-specific and had been isolated from adult *Ae. albopictus* before*,* with Aedes phasmavirus being detected in all life stages of the mosquito (Manni and Zdobnov 2020; Shi et al. 2020). In addition, both *Ae. albopictus* sexes harboured RNA similar to Barstukas virus (97.91–100% p.i., 83–524 bp, 5 contigs) and Guapiaçu virus (98.50–98.83% p.i., 133–170 bp, 2 contigs) which had previously been identified in adult *Aedes* mosquitoes (Batson et al. 2021). RNA suggesting High Island virus (97.69–98.70% p.i., 120–386 bp, 3 contigs), Usinis virus (99.74–100%, p.i., 118–390 bp, 6 contigs) and Wenzhou sobemo–like virus (96.47–100% p.i., 31–1677 bp, 750 contigs) was also found in both female and male *Ae. albopictus*. High Island virus had formerly been detected in adult mosquitoes of the species *Psorophora ciliata* (Sadeghi et al. 2017), Usinis virus in adult *Ae. aegypti* and *Ae. albopictus* (Batson et al. 2021) and Wenzhou sobemo-like virus in adult *Ae. albopictus* (Kubacki et al. 2020).

In addition to the RNA of these viruses, RNA with high identities to various bacteria was identified. Among others, RNA largely matching *Acinetobacter baumannii* (97.50–100% p.i., 40–170 bp, 3 contigs), *A. johnsonii* (98.76–100% p.i., 150–188 bp, 7 contigs), *Elizabethkingia anophelis* (99.12–100% p.i., 90–480 bp, 7 contigs), *Escherichia coli* (97.37–100% p.i., 53–167 bp; 14 contigs) and *W. pipientis* (96.91–100% p.i., 33–1052 bp, 204 contigs) was found in both female and male *Ae. albopictus*. *Acinetobacter baumannii* and *A. johnsonii* are human pathogens and had previously been reported from adult *Ae. albopictus* (Seifert et al. 1993; Minard et al. 2013), *A. baumannii* also from lice and fleas (Kempf et al. 2012)*.* The ubiquitous *E. coli* is a potentially pathogenic (Kaper et al. 2004), widely spread intestinal bacterium of humans and other vertebrates, which had been recognised in adult *Anopheles funestus* and *An. gambiae* before (Straif et al. 1989). *Elizabethkingia anophelis* has emerged as a human pathogen in Africa and Asia (Lau et al. 2016) and had previously been detected in adult *An. gambiae* (Kämpfer et al. 2011). *Wolbachia pipientis* is a widely distributed essential bacterial symbiont of mosquitoes, which had frequently been described from *Ae. albopictus* (e.g. Wiwatanaratanabutr 2013; Park et al. 2016) and other mosquito species (e.g. Kittayapong et al. 2000).

Finally, RNA corresponding to that of *Candida sake* (100% p.i., 104–189 bp, 3 contigs) was detected in both *Ae. albopictus* females and males in this study. The fungus belongs to a genus widely distributed in arthropods but has also been extracted from the oral cavity of HIV-positive humans (Hoegl et al. 1998).

In addition to RNA fragments suggesting the same microbial species to occur in both *Ae. albopictus* females and males, RNA fragments referring to some bacteria were found in one mosquito sex only (Supplementary Tables 1 and 2). These include those of the bacteria *A. dispersus* (99.72% p.i., 422 bp, 1 contig)*, A. oleivorans* (100% p.i., 1378 bp, 2 contigs)*, Chryseobacterium aureum* (99.11% p.i., 676 bp, 1 contig)*, C. indoltheticum* (100% p.i., 602–686 bp, 3 contigs)*, C. scophthalmum* (99.79% p.i., 470 bp, 1 contig)*, Leclercia adecarboxylata* (100% p.i., 96–546 bp, 6 contigs), *Limnobacter humi* (100% p.i., 106 bp, 1 contig)*, Serratia marcescens* (100% p.i., 80 bp, 1 contig) and *Zooglea resiniphila* (100% p.i., 56 bp, 1 contig) in the males (Table 1). *Acinetobacter dispersus* can be frequently found on human skin and in water and soil (Kang et al. 2011; Nemec et al. 2016). *Acinetobacter oleivorans* had been detected in soil (Kang et al. 2011) and *C. aureum* in river water in Korea (Lee et al. 2019). *Chryseobacterium indoltheticum* is a widespread bacterium occurring in soil and water which may be pathogenic to humans (Calderón et al. 2011), and *C. scophthalmum* is a fish pathogen (Shahi et al. 2018)*. Leclercia adecarboxylata* had previously been documented in other insects such as the potato beetle *Leptinotarsa decemlineata* (Muratoglu et al. 2009) and is also considered potentially pathogenic for humans (Hess et al. 2008). By contrast, *L. humi* had been recognised from humus soil (Nguyen and Kim 2017). Another human pathogen similar to RNA which was found in male *Ae. albopictus* was *S. marcescens* (Hejazi and Falkiner 1997). This bacterium had been detected in adult *An. sinensis* mosquitoes previously (Bai et al. 2019) and might become a problem in mosquito laboratory colonies (Seitz et al. 1987). *Zooglea resiniphila* had been found in activated sludge (Gao et al. 2018).

RNAs with similarity to some bacterial species were identified in the female *Ae. albopictus* of this study but not in the males (Table 1). These include *Acidovorax avena* (100% p.i., 121 bp, 1 contig), *Acinetobacter tandoii* (100% p.i., 558 bp, 1 contig)*, Aeromonas hydrophila* (100% p.i., 33–1012 bp, 5 contigs), *Arthrobacter woluwensis* (100% p.i., 120–411 bp, 4 contigs)*, Hydrogenophaga pseudoflava* (100% p.i., 176–189 bp, 2 contigs)*, Micrococcus luteus* (100% p.i., 118–140 bp, 2 contigs)*, Paracoccus yeei* (100% p.i., 126–168 bp, 2 contigs) and *Pseudomonas luteola* (100% p.i., 137–198 bp, 2 contigs). *Acidovorax avenea* is a plant-pathogenic bacterium (Walcott and Gitaitis 2000), whereas *A. tandoii* had been detected in termites (van Dexter and Boopathy 2019). *Aeromonas hydrophila* is a pathogen of many different vertebrates including humans (Emerson and Norris 1905; Wohlgemut et al. 1970; Agger et al. 1985), which can naturally be found in water habitats (Hazen et al. 1978). *Arthrobacter woluwensis* is a potential human pathogen, which can cause endocarditis, among other symptoms (Bernasconi et al. 2004; Li et al. 2021)*. Hydrogenophaga pseudoflava* had previously been detected in the midgut of adult *An. gambiae* (Straif et al. 1989). RNA fragments suggesting another potential human pathogen, which had led to human meningitis in the past, is *M. luteus* (Fosse et al. 1985)*. Paracoccus yeei*, on the other hand, is a human bacterial pathogen, which had formerly been isolated from the salivary glands of adult *Ae. aegypti* (Balaji et al. 2021) and can lead to human dialysis-related peritonitis (Arias and Clark 2019). *Pseudomonas luteola* is another fish pathogen, which can cause, for example, meningitis and wound infection in immunocompromised humans (Kostmann et al. 1990; Altinok et al. 2007)*.*

In addition to RNAs with high identities to the above bacteria found in only one sex of *Ae. albopictus*, RNA with a high identity to the fungus *Conidiobolus coronatus*, which has a human-pathogenic potential (Fischer et al. 2008), could be detected in the female mosquitoes.

## Discussion

The mosquitoes in this study were pooled from seven sites within Germany known to be populated by *Ae. albopictus*. Since the tiger mosquito is controlled in Germany by Bti (*Bacillus thuringiensis israelensis*) larvicide as soon as local reproduction is detected (Becker et al. 2017, 2022), the finding of larvae is difficult and was limited in the framework of this study. Due to the pooling of the collected samples, no statement can be made about the geographical origin of the microorganisms or the individual colonisation of *Ae. albopictus* specimens. Moreover, all viruses and microorganisms referred to in this study were identified exclusively by their RNAs and the alignment of those with sequences in the used databases. Therefore, it is not known whether exactly these species were present or other (unknown) species with closely related RNA sequences, whether they were viable viruses, living symbionts or similar and able to replicate/multiply in *Ae. albopictus*, and whether they were arbitrarily taken up from the environment and had no additional correlation to the mosquitoes.

As adult mosquitoes emerged from collected larvae were tested here, and no food sources whatsoever had been offered to the investigated adults, the detected RNAs, or the microbes characterised by them, must be supposed to have been transmitted transstadially from mosquito larva to pupa and through metamorphosis to adult. During metamorphosis, the midgut of mosquitoes is transformed and the digestive cells are histolised (Fernandes et al. 2014). The survival of microorganisms or the persistence of RNA, respectively, must therefore be supposed to be possible only intracellularly or with certain mechanisms of adapted symbionts. To clarify this and check for the viability of microorganisms, instead of mere RNA, cultivation attempts are necessary, but were not carried out in this study.

As we studied the microbial RNA metagenome of complete mosquitoes, it cannot be determined in which organ or tissue the found RNAs had been localised. Principally, the composition of the bacterial fauna in different organs of a mosquito can be very variable, and some bacteria colonise several organs in the mosquito at the same time (Gao et al. 2020). The tropism of microbes might give information about their migration paths in the mosquito, or about transmissibility from mosquito parent to offspring or mosquito female to blood host. For the latter, the emergence of the microorganism in the mosquito salivary glands would be requisite (Anderson et al. 2010). Whether this was the case for the potential human pathogens RNAs found were suggestive of, such as *A. baumannii*, *E. coli* or *A. hydrophila*, cannot be determined retrospectively. In addition to the localisation of the microorganisms in the mosquito, the pathogen load which was also not determined in this study might be decisive for a mosquito to become a vector. Thus, the mere presence of a pathogen in the mosquito might not be sufficient for transmission (Beerntsen et al. 2000).

The presence in the microbiome of certain bacteria is beneficial to the mosquito. For example, *A. baumannii* and *A. johnsonii* improve blood digestion and nectar assimilation in *Ae. albopictus* (Minard et al. 2013). However, the influence on mosquito development, reproduction and physiology of most microorganisms found in the microbiome is largely unknown.

The most common phyla ever found in the microbiome of adult *Ae. albopictus* include Proteobacteria, Bacteroidetes, Firmicutes and Actinobacteria (Mancini et al. 2018). The most common bacterial genera found in *Aedes, Anopheles* and *Culex* species are *Enterobacter, Escherichia, Klebsiella, Pseudomonas* and *Serratia* as detected in mosquitoes from the USA, England and India (Demaio et al. 1996; Touré et al. 2000; Pidiyar 2002). RNA with identities to all four bacterial phyla as well as to all five genera were found in the *Ae. albopictus* samples from Germany. In addition, RNA fragments indicating viruses and fungi, such as Riboviria and Ascomycota, were identified. In summary, RNAs with identities to a high number of microorganisms were detected in the German *Ae. albopictus* some of which represent microorganisms already described from this mosquito species previously, such as *W. pipientis*, *A. baumannii* or Usinis virus (Minard et al. 2013; Wiwatanaratanabutr 2013; Batson et al. 2021). Some other viruses or microbial species suggested by the RNA analysis in this study, such as High Island virus, Guapiaçu virus and *E. anophelis*, have not been detected in *Ae. albopictus* before, but in other mosquito species and other invertebrates (Kämpfer et al. 2011; Sadeghi et al. 2017; Batson et al. 2021; Oliveira Ribeiro et al. 2021). Furthermore, RNA fragments suggesting microorganisms previously not described from mosquitoes at all, such as *L. humi*, *Z. resiniphila* and *C. aureum*, were found.

It has also been shown that the midgut microbiome of adult mosquitoes may reduce a mosquito’s susceptibility to pathogens (Dong et al. 2009; Bahia et al. 2014) and have a general influence on its vector competence (Dodson et al. 2014; Jupatanakul et al. 2014). It can thus be harnessed by manipulating its microorganisms to artificially reduce vector competence. In culture, for example, *E. coli* was genetically modified in order to express two surface molecules that suppress the development of *Plasmodium berghei*. Unfortunately, *E. coli* had difficulties in colonising the mosquitoes and disappeared from their midgut shortly after infection (Riehle et al. 2007). In addition, there are studies that show that an infection of mosquitoes with *Wolbachia* leads to a strong inhibition of the development of potential pathogens. The infection with *Wolbachia* of the wMel strain, for example, leads to a highly reduced replication of dengue virus in *Ae. aegypti*. The reason for this seems to be the *Wolbachia*-linked upregulation of the immune system of the mosquito (Blagrove et al. 2012). Such a way of influencing vector competence is certainly also possible with the help of other organisms from the microbiome.

Since insecticides and physical measures are often inefficient tools for mosquito control (Bourtzis et al. 2016; Pang et al. 2017; Flores and O'Neill 2018), *W. pipientis* is also exploited for innovative biological control by manipulating mosquito reproduction. *Wolbachia* infection can lead to the feminisation of genetically male insects, to the killing of male siblings by females or cytoplasmic incompatibility, which ensures that females can successfully mate only with males harbouring the same *Wolbachia* strain (Werren et al. 2008).

Efficient control tools are particularly important in areas where mosquitoes serve as vectors of human disease agents such as Zika virus, yellow fever virus and malaria parasites. Knowledge about the natural occurrence of microorganisms in mosquitoes can therefore contribute to developing and designing new forms of vector control.

It is difficult to explain the differences in the RNA presence between female and male *Ae. albopictus* in this study. Individuals of both genders were collected from the same breeding sites of the seven locations, so differences between the microbial RNA cannot be attributed to developmental conditions. However, RNA with high identities to 25 species of microorganisms were found in the males but not in the females, and RNA with high identities to 29 species of microorganisms were found in the females but not in the males, with clear differences in the distribution of microorganism to kingdoms (in females, most contigs were assigned to bacteria, whereas in males most contigs were assigned to viruses). For example, RNA fragments arguing for plants were found in the pool of males, but not in the pool of females. That sex has an influence on the microbiome has been shown in previous studies (Chen et al. 2020). Also, Rani et al. (2009) detected *Chryseobacterium*, *Pseudomonas* and *Serratia* species only in females of *Anopheles stephensi*. One possible explanation for the differences might be owing to the limited number of individuals examined, with such differences becoming smaller the more individuals are studied per site. Another explanation might be that female and male larvae in fact have different food preferences and therefore take up different microorganisms with their food, but this is mere speculation and cannot be substantiated. Although it is known that larvae consume a wide range of food (Gimnig et al. 2002; Ye-Ebiyo et al. 2003) and have different feeding preferences depending on species (Merritt et al. 1992), no data exist about different feeding preferences of female and male larvae of the same species.

Especially in cases of a low percent identity and a low query coverage of the found contig sequences with sequences in GenBank, results have to be considered carefully with regard to the occurrence of the respective microorganisms in German *Ae. albopictus*. This applies, for example, to *Kocuria rhizophila*. RNA found in female *Ae. albopictus* matched this soil bacterium with a percent identity of 85.43% only and was registered with only one read. Due to the low percent identity, it is unlikely that the detected RNA belonged to exactly this species. Also, in the case of very short sequence lengths, the linked microorganisms must be viewed critically. This was the case for *Wuchereria bancrofti*-RNA where the sequence length was 45 bp only. Although percent identity and query coverage were both close to 100%, the probability of the RNA belonging to another organism, possibly a filarial species not described so far, is very high. Hitherto, *W. bancrofti* has not been reported from *Ae. albopictus*, and its geographic distribution range is restricted to subtropical and tropical regions (Service 2001).

Possible contamination must also be considered and, in fact, has already been described in similar studies. Genera such as *Flavobacterium*, *Micrococcus*, *Microbacterium*, *Chryseobacterium*, *Neskia* and *Acidovorax* have been found contaminating laboratory reagents like DNA extraction kits (Salter et al. 2014). In this study, *Flavobacterium*, *Micrococcus*, *Chryseobacterium* and *Acidovorax* could be detected in both sexes of German *Ae. albopictus*, *Neskia* only in females.

## Conclusion

The microbiome of vector species such as *Ae. albopictus* holds much potential for the development of efficient control measures and the reduction of vector competence. However, the influence of many microorganisms on the mosquito is still largely unexplored.

Studying the composition of a mosquito’s microbiome is difficult, as it is influenced by many factors and can vary considerably both within a species and between species. It is necessary to investigate the influences of the microbiome in more detail and to examine its diversity in a large number of mosquito microbiomes.

To clarify the question of which influence the microorganisms of the microbiome have on the mosquito, it would be helpful to cultivate the microorganisms. However, cultivation is only possible with a small number of species of the microorganisms detected so far. Only with detailed knowledge about the composition and influence of the microbiome on the mosquito can these microorganisms be used for innovative approaches to vector and disease management. The study demonstrates that the microbiome of German *Ae. albopictus* is comprehensive and might be worth further investigations.

## Supplementary Information

Below is the link to the electronic supplementary material.

Supplementary file1 (DOCX 83 KB)

## Data Availability

Data supporting the conclusions of this article are included within the article and its supplementary tables.
