# Supplementary material for: The microbial RNA metagenome of Aedes albopictus (Diptera: Culicidae) from Germany
Source: Parasitol Res. 2022 Jul 20;121(9):2587–99. doi: 10.1007/s00436-022-07576-7 (PMC9378336; doi:10.1007/s00436-022-07576-7)
Supplement: Supplementary file 1 — Supplementary file1 (DOCX 83 KB) [file 436_2022_7576_MOESM1_ESM.docx]

**Supplementary Table 1**

| Species | Phylum | Family | Sequence length [bp] | Number of sequences | Query cover [%] | Percent identity [%] | Remarks |
| --- | --- | --- | --- | --- | --- | --- | --- |
| Virus | | | | | | | |
| Aedes albopictus anphevirus * | Riboviria | unclassified Anphevirus | 25-50  50-100  100-150  150-200  200-250  250-350  350-450  450-1000 | 265 | 100 | 100  99.69  96.84  94.63  89.23 | Insect-specific virus. Previously detected in adult *Ae. albopictus* (Manni and Zdobnov 2020). |
| Aedes phasmavirus * | Riboviria | unclassified Phasmaviridae | 25-50  50-100  150-200  350-450 | 5 | 100 | 100  98.39  96.57 | Previously detected in adult *Ae. albopictus* (Shi et al. 2020). |
| Aedes phasmavirus or Makri bunya-like virus | Riboviria | unclassified Phasmaviridae | 450-1000 | 1 | 100 | 96.79 | */* |
| Barstukas virus * | Riboviria | unclassified Riboviria | 50-100  100-150  150-200  450-1000 | 4 | 100 | 100  98.68 | Previously detected in adult mosquitoes (Batson et al. 2021). |
| Guangzhou sobemo-like virus * | Riboviria | Solemoviridae | 50-100 | 1 | 100 | 98.55 | Previously detected in adult *Ae. albopictus* (Shi et al. 2020). |
| Guangzhou-sobemo like virus or Nea chili luteo-like virus | Riboviria or  Orthornavirae | Solemoviridae or  Luteoviridae | 50-100  150-200 | 2 | 100 | 100  95.18 | / |
| Guapiacu-virus * | Riboviria | Flaviviridae | 350-450 | 1 | 100 | 98.83 | Insect-specific virus. Previously detected in Brazil in adult *Ae. terrens* and *Ae. scapularis* (Batson et al. 2021; Oliveira Ribeiro et al. 2021). |
| High Island virus * | Riboviria | Reoviridae | 350-450 | 1 | 100 | 98.70 | Previously detected in adult mosquitoes and other invertebrates (Sadeghi et al. 2017). |
| Nea chili luteo-like virus | Riboviria | Luteoviridae | 150-200 | 1 | 100 | 98.43 | Previously detected in adult *Ae. albopictus* (Konstantinidis et al. 2021). |
| Usinis virus * | Ribovaria | Unclassified  Riboviria | 150-200  350-450 | 2 | 100 | 100  99.74 | Previously detected in adult *Ae. albopictus* (Batson et al. 2021). |
| Wenzhou sobemo-like virus * | Riboviria | unclassified Riboviria | 25-50  50-100  100-150  150-200  350-450  450-1000  1000-2200 | 733 | 100 | 100 | Previously detected in adult *Ae. albopictus* (Kubacki et al. 2020). |
| Wenzhou sobemo-like virus or Guangzhou sobemo-like virus * | Riboviria | Unclassified  Riboviria or  Solemoviridae | 25-50  50-100  350-450  450-1000 | 4 | 100 | 100  97.63 | / |
| Wenzhou sobemo-like virus or Nea chili luteo-like virus | Riboviria | unclassified Riboviria or  Luteoviridae | 150-200  450-1000 | 4 | 100  96 | 98.77  97.87  96.92 | / |
| Bacteria | | | | | | | |
| *Acidovorax* sp. | Proteobacteria | Comamonadaceae | 25-50 | 1 | 100 | 100 | / |
| *Acidovorax* sp. or *Curvibacter* sp*.* | Proteobacteria | Comamonadaceae | 100-150 | 2 | 100 | 99.3 | / |
| *Acidovorax* sp. or *Ralstonia* sp. | Proteobacteria | Comamonadaceae or Burkholderiaceae | 350-450 | 1 | 100 | 100 | / |
| *Acinetobacter baumannii* * | Proteobacteria | Moraxellaceae | 50-100 | 1 | 100 | 97.5 | Human pathogen, previously detected in adult *Ae. albopictus* (Minard et al. 2013). |
| *Acinetobacter calcoaceticus* or *lactucae* | Proteobacteria | Moraxellaceae | 350-450 | 1 | 100 | 99.26 | / |
| *Acinetobacter dispersus* | Proteobacteria | Moraxellaceae | 350-450 | 1 | 100 | 99.72 | Previously detected on human skin and wounds, water and soil (Nemec et al. 2016). |
| *Acinetobacter johnsonii** | Proteobacteria | Moraxellaceae | 100-150  150-200 | 6 | 100 | 100  99.12 | Previously detected in adult *Ae. albopictus* and other mosquito species; may cause bloodstream infections in humans due to vascular catheters (Seifert et al. 1993; Minard et al. 2013). |
| *Acinetobacter oleivorans* | Proteobacteria | Moraxellaceae | 50-100  1000-2200 | 2 | 100 | 100  98.11 | Previously detected in the rhizosperic zone of *Zea mays* (Uniyal et al. 2016). |
| *Acinetobacter seifertii* | Proteobacteria | Moraxellaceae | 50-100 | 1 | 100 | 98.51 | Part of the *A. calcoaceticus*-*A. baumannii* complex; previously detected in human clinical samples (Cerezales et al. 2018). |
| *Acinetobacter seifertii* or *calcoaceticus* | Proteobacteria | Moraxellaceae | 150-200 | 1 | 100 | 100 | / |
| *Acinetobacter soli* | Proteobacteria | Moraxellaceae | 50-100 | 1 | 100 | 100 | Previously detected in soil (Kim et al. 2008). |
| *Acinetobacter* sp. * | Proteobacteria | Moraxellaceae | 25-50  50-100  100-150  150-200  350-450  450-1000 | 16 | 100 | 100  99.25  98.32 | / |
| *Aeromonas* sp. * | Proteobacteria | Aeromonadaceae | 50-100  100-150  150-200  350-450 | 5 | 100 | 100  99.35  97.89 | / |
| *Aquabacterium olei* | Proteobacteria | Comamonadaceae | 150-200 | 1 | 100 | 97.81 | Previously detected in oil-contaminated soil from Korea (van Pham et al. 2015). |
| *Aquirufa nivalisilvae* | Bacteroidetes | Cytophagaceae | 50-100  150-200  200-250  350-450 | 4 | 100 | 97.95  93.88  85.00  84.46 | Previously detected in fresh water (Pitt et al. 2019). |
| *Aquitalea* sp. | Proteobacteria | Chromobacteriaceae | 450-1000 | 1 | 100 | 98.66 | / |
| *Cellvibrio* sp. | Proteobacteria | Cellvibrionaceae | 150-200 | 1 | 100 | 99.49 | / |
| *Chromobacterium* sp. * | Proteobacteria | Neisseriaceae | 1000-2200 | 1 | 100 | 95.89 | / |
| *Chryseobacterium aureum* | Bacteroidetes | Flavobacteriaceae | 450-1000 | 1 | 100 | 99.11 | Previously detected in river in Korea (Lee et al. 2019a). |
| *Chryseobacterium gleum* or *arthrosphaera* | Bacteroidetes | Flavobacteriaceae | 150-200 | 1 | 100 | 100 | / |
| *Chryseobacterium indoltheticum* | Bacteroidetes | Flavobacteriaceae | 150-200  450-1000 | 3 | 100 | 99.56  100 | Previously detected in marine mud, human pathogen (Calderón et al. 2011). |
| *Chryseobacterium nakagawai* or *arthrosphaera* | Bacteroidetes | Flavobacteriaceae | 150-200 | 1 | 100 | 100 | / |
| *Chryseobacterium scophthalmum* | Bacteroidetes | Flavobacteriaceae | 450-1000 | 1 | 100 | 99.79 | Fish pathogen (Shahi et al. 2018). |
| *Chryseobacterium* sp. | Bacteroidetes | Flavobacteriaceae | 50-100  150-200  350-450  450-1000 | 7 | 100 | 100  99.20  98.59  97.86 | / |
| *Chryseobacterium* sp. or *Elizabethkingia* sp. | Bacteroidetes | Flavobacteriaceae or  Weeksellaceae | 50-100 | 1 | 100 | 100 | / |
| *Chryseobacterium* sp. or *Epilithonimonas* sp. | Bacteroidetes | Flavobacteriaceae | 350-450 | 1 | 100 | 98.38 | / |
| *Cloacibacterium normanense* | Bacteroidetes | Flavobacteriaceae | 150-200 | 1 | 100 | 100 | Previously detected in wastewater (Allen et al. 2006). |
| *Comamonas serinivorans* | Proteobacteria | Comamonadaceae | 50-100 | 1 | 100 | 100 | Previously detected in wheat straw compost in China (Zhu et al. 2014). |
| *Comamonas* sp. | Proteobacteria | Comamonadaceae | 50-100 | 1 | 100 | 100 | / |
| *Curvibacter* sp. | Proteobacteria | Comamonadaceae | 150-200 | 1 | 100 | 100 | / |
| *Dechloromonas* sp. | Proteobacteria | Azonexaceae | 50-100  100-150 | 1 | 100 | 100 | / |
| *Delftia* sp. * | Proteobacteria | Comamonadaceae | 50-100  100-150 | 2 | 100 | 100  97.97 | / |
| *Diaphorobacter* sp. or *Acidovorax* sp. | Proteobacteria | Comamonadaceae | 100-150 | 1 | 100 | 100 | / |
| *Elizabethkingia anopheles* * | Bacteroidetes | Weeksellaceae | 50-100  100-150  350-450  450-1000 | 6 | 100  99.12 | 100 | Human pathogen; previously detected in adult *An. gambiae* (Kämpfer et al. 2011). |
| *Elizabethkingia* sp. | Bacteroidetes | Weeksellaceae | 25-50  350-450 | 2 | 100 | 100  99.74 | / |
| *Enterobacter hormaechei* or *cloacae* | Proteobacteria | Enterobacteriaceae | 150-200 | 1 | 100 | 100 | / |
| *Enterobacter roggenkampii* or *Leclercia adecarboxylata* | Proteobacteria | Enterobacteriaceae | 1000-2200 | 1 | 100 | 98.51 | / |
| *Enterobacter* sp. | Proteobacteria | Enterobacteriaceae | 50-100 | 1 | 100 | 100  98.41 | / |
| *Enterobacter* sp. or *Leclercia* sp. | Proteobacteria | Enterobacteriaceae | 350-450 | 1 | 100 | 99.48 | / |
| *Escherichia coli* * | Proteobacteria | Enterobacteriaceae | 50-100  100-150  150-200 | 6 | 100 | 100  99.30  97.37 | Intestinal bacterium (Kaper et al. 2004); previously detected in adult *An. funestus* (Straif et al. 1989). |
| *Exiguobacterium sp.* | Firmicutes | Bacillaceae | 150-200 | 1 | 100 | 100 | / |
| *Flavobacterium* sp. * | Bacteroidetes | Flavobacteriaceae | 100-150  150-200 | 2 | 100 | 100  99.17 | / |
| *Flectobacillus* sp. | Bacteroidetes | Spirosomaceae | 100-150 | 1 | 97 | 100 | / |
| *Gemmobacter fontiphilus* | Proteobacteria | Rhodobacteraceae | 150-200 | 1 | 100 | 98.34 | Previously detected in fresh water spring (Chen et al. 2013). |
| *Hydrogenophaga* sp. * | Proteobacteria | Comamonadaceae | 50-100 | 1 | 100 | 100 | / |
| *Leclercia adecarboxylata* | Proteobacteria | Enterobacteriaceae | 50-100  100-150  150-200  450-1000 | 6 | 100 | 100 | Human pathogen (Hess et al. 2008). |
| *Limnobacter humi* | Proteobacteria | Burkholderiaceae | 100-150 | 1 | 100 | 100 | Previously detected in humus soil (Nguyen and Kim 2017). |
| *Limnobacter* sp. * | Proteobacteria | Burkholderiaceae | 50-100 | 1 | 100 | 100 | / |
| *Limnohabitans* sp. | Proteobacteria | Comamonadaceae | 50-100 | 1 | 100 | 100 | / |
| *Mesorhizobium* sp. | Proteobacteria | Phyllobacteriaceae | 50-100 | 1 | 100 | 100 | / |
| *Methylibium petroleiphilum* | Proteobacteria | Comamonadaceae | 50-100 | 1 | 100 | 98.55 | Previously detected in a filter for sewage treatment plants (Schmidt et al. 2008). |
| *Microbacterium* sp. * | Actinobacteria | Mycobacteriaceae | 150-200  350-450 | 3 | 100 | 100  93.95 | / |
| *Paucibacter* sp. * | Proteobacteria | Burkholderiales genera inc. sed. | 50-100 | 1 | 100 | 100 | / |
| *Paucibacter* sp. or *Vitreoscilla filiformis* | Proteobacteria | Burkholderiales genera inc. sed. or  Neisseriaceae | 50-100 | 1 | 100 | 100 | / |
| *Pelomonas* sp. | Proteobacteria | Comamonadaceae | 50-100 | 1 | 100 | 100 | / |
| *Pseudomonas* sp. * | Proteobacteria | Pseudomonadaceae | 50-100  350-450 | 3 | 100 | 100  99.16 | / |
| *Rhizobium selenitireducens* | Proteobacteria | Rhizobiaceae | 150-200 | 1 | 100 | 100 | Previously detected in a bioreactor (Hunter et al. 2007). |
| *Runella slithyformis* | Bacteroidetes | Cytophagaceae | 150-200 | 1 | 100 | 96.43 | Previously detected in fresh water (Larkin and Williams 1978). |
| *Runella* sp. | Bacteroidetes | Cytophagaceae | 100-150 | 1 | 100 | 100 | / |
| *Serratia marcescens* | Proteobacteria | Enterobacteriaceae | 50-100 | 1 | 100 | 100 | Human pathogen; previously detected in *Anopheles* mosquitoes (Hejazi and Falkiner 1997; Bai et al. 2019). |
| *Serratia* sp. | Proteobacteria | Enterobacteriaceae | 100-150 | 1 | 100 | 100 | / |
| *Solimonas* sp. | Proteobacteria | Sinobacteraceae | 150-200 | 1 | 100 | 98.74 | / |
| Uncultured actinobacterium | Actinobacteria | — | 50-100 | 1 | 100 | 100 | / |
| Uncultured bacterium * | — | — | 50-100  100-150  150-200  200-250  350-450 | 14 | 100  80 | 100  99.09  98.98  96.97  94.29 | / |
| Uncultured beta proteobacterium | Proteobacteria | — | 450-1000 | 1 | 100 | 98.59 | / |
| Uncultured gamma proteobacterium | Proteobacteria | — | 100-150 | 1 | 100 | 98.94 | / |
| Uncultured Nocardioides sp. | Actinobacteria | Nocardioidaceae | 100-150 | 1 | 100 | 97.66 | / |
| Uncultured Sphingobacteriales bacterium | Bacteriodetes | Sphingobacteriales | 50-100 | 1 | 100 | 90.20 | / |
| *Undibacterium piscinae* or *parvum* | Proteobacteria | Oxalobacteraceae | 150-200 | 1 | 100 | 99.36 | / |
| *Undibacterium* sp. | Proteobacteria | Oxalobacteraceae | 150-200 | 1 | 100 | 100 | / |
| *Variovorax* sp. | Proteobacteria | Comamonadaceae | 25-50 | 1 | 100 | 97.92 | / |
| *Variovorax* sp. or *Hydrogenophaga* sp. | Proteobacteria | Comamonadaceae | 150-200 | 1 | 100 | 100 | / |
| *Vogesella* sp. * | Proteobacteria | Chromobacteriaceae | 50-100  450-1000 | 2 | 100 | 100  97.43 | / |
| *Wolbachia pipientis* * | Proteobacteria | Anaplasmataceae | 25-50  50-100  100-150  150-200  350-450  450-1000  1000-2200 | 186 | 100  96  95 | 100  99.73  96.91 | Previously detected in adult *Ae. albopictus* (Wiwatanaratanabutr 2013). |
| *Wolbachia* sp. * | Proteobacteria | Anaplasmataceae | 150-200 | 2 | 93.00 | 100  97.50 | / |
| *Zooglea resiniphila* | Proteobacteria | Rhodocyclaceae | 50-100 | 1 | 100 | 100 | Previously detected in activated sludge (An et al. 2016). |
| *Zoogloea* sp. | Proteobacteria | Rhodocyclaceae | 150-200 | 1 | 100 | 100 | / |
| Eukaryotes | | | | | | | |
| *Brassica rapa* or *napus* | Brassicales | Brassicaceae | 50-100 | 2 | 100 | 100 | Food turnip or rapeseed. |
| *Candida sake* * | Ascomycota | incertea sedis | 150-200 | 1 | 100 | 100 | Previously detected in oral cavity of HIV-positive people (Hoegl et al. 1998). |
| Different plants | — | — | 100-150  150-200  450-1000 | 9 | 100 | 100  99.49  98.19 | / |
| *Dinobryon* sp. * | Stramenopiles | Dinobryonaceae | 100-150 | 1 | 100 | 100 | Freshwater alga. |
| *Keratosa* sp. | Metazoa | Demospongiae | 25-50 | 1 | 100 | 100 | / |
| *Ostreococcus lucimarinus* | Chlorophyta | Bathycoccaceae | 200-250 | 1 | 100 | 82.99 | Green alga; previously detected in seawater (Derelle et al. 2015). |
| *Paecilomyces* sp. | Eurotiomycetes | Trichocomaceae | 50-100 | 1 | 100 | 98.15 | / |
| *Polymyxa graminis* | Endomyxa | Plasmodiophoridae | 50-100 | 1 | 100 | 100 | Parasite of plant roots; can transmit plant viruses (Kanyuka et al. 2003). |
| *Salarias fasciatus* | Craniata | Blenniidae | 50-100 | 1 | 100 | 97.96 | Fish species. |
| Uncultured eukaryote | — | — | 25-50 | 1 | 100 | 100 | / |
| Uncultured fungus * | — | — | 50-100  100-150 | 7 | 100  80 | 100  98.46  97.22 | / |

**Supplementary Table 2**

| Species | Phylum | Family | Sequence length [bp] | Number of sequences | Query cover [%] | Percent identity [%] | Remarks |
| --- | --- | --- | --- | --- | --- | --- | --- |
| Viruses | | | | | | | |
| Aedes albopictus anphevirus * | Riboviria | unclassified Anphevirus | 50-100  100-150  150-200  250-350  350-450 | 16 | 100 | 100 99.26  95.60 | Insect specific virus; previously detected in adult *Ae. albopictus* (Manni and Zdobnov 2020). |
| Aedes phasmavirus * | Riboviria | unclassified Phasmaviridae | 100-150  150-200  200-250  250-350 | 8 | 100 | 99.44  97.88  98.53 | Previously detected in adult *Ae. albopictus* (Shi et al. 2020). |
| Barstukas virus * | Riboviria | unclassified Riboviria | 450-1000 | 1 | 100 | 96.91 | Previously detected in adult mosquitoes (Batson et al. 2021). |
| Barstukas virus or Aedes phasmavirus | Riboviria | unclassified Riboviria or Phasmaviridae | 50-100  100-150 | 2 | 100 | 100  98.04 | / |
| Guangzhou sobemo-like virus * | Riboviria | Solemoviridae | 150-200 | 1 | 100 | 93.40 | Previously detected in adult *Ae. albopictus* (Shi et al. 2020). |
| Guapiacu-virus * | Riboviria | Flaviviridae | 100-150 | 1 | 100 | 98.50 | Insect-specific virus; previously detected in adult *Ae. terrens* and *Ae. scapularis* (Oliveira Ribeiro et al. 2021). |
| High Island virus * | Riboviria | Reoviridae | 150-200, 250-350 | 2 | 100 | 98.33  97.69 | Previously detected in adult mosquitoes and other invertebrates (Sadeghi et al. 2017). |
| Hypsignathus monstrosus tombus-like virus | Riboviria | Tombusviridae | 1000-2200 | 1 | 100 | 77.19 | Previously detected in bats (Bennett et al. 2019). |
| Usinis virus * | Riboviria | Unclassified Riboviria | 100-150  150-200 | 4 | 100 | 100  99.15 | Previously detected in adult *Ae. albopictus* (Batson et al. 2021). |
| Wenzhou sobemo-like virus * | Riboviria | unclassified Riboviria | 25-50  50-100  100-150  350-450  450-1000  1000-2200 | 17 | 100 | 100  99.41  98.73  97.33  96.47  91.45 | Previously detected in adult *Ae. albopictus* (Kubacki et al. 2020). |
| Wenzhou sobemo-like virus or Guangzhou sobemo-like virus * | Riboviria | unclassified Riboviria or Solemoviridae | 50-100  100-150 | 4 | 100 | 100 | / |
| Bacteria | | | | | | | |
| *Acidovorax avena* | Proteobacteria | Comamonadaceae | 100-150 | 1 | 100 | 100 | Plant pathogen (Walcott und Gitaitis 2000). |
| *Acidovorax* sp. or *Variovorax* sp. | Proteobacteria | Comamonadaceae | 100-150  150-200 | 2 | 100 | 100 | / |
| *Acinetobacter baumanii* * | Proteobacteria | Moraxellaceae | 50-100  150-200 | 2 | 100 | 100  97.65 | Human pathogen; previously detected in adult *Ae. albopictus* (Minard et al. 2013). |
| *Acinetobacter johnsonii ** | Proteobacteria | Moraxellaceae | 150-200 | 1 | 100 | 98.76 | Human pathogen; previously detected in adult *Ae. albopictus* and other mosquitoes (Seifert et al. 1993; Minard et al. 2013). |
| Acinetobacter sp. * | Proteobacteria | Moraxellaceae | 25-50  100-150  450-1000 | 6 | 100 | 100  97.20  69.91 | / |
| *Acinetobacter tandoii* | Proteobacteria | Moraxellaceae | 450-1000 | 1 | 100 | 100 | Previously detected in termite (van Dexter und Boopathy 2019). |
| *Acinetobacter tjernbergiae* | Proteobacteria | Moraxellaceae | 350-450 | 1 | 100 | 99.29 | Previously detected in activated sludge (Carr et al. 2003). |
| *Aeromonas hydrophila* | Proteobacteria | Aeromonadaceae | 25-50  200-250  450-1000  1000-2000 | 5 | 100 | 100  99.35 | Pathogen of humans and many animals; previously detected in water habitats (Emerson and Norris 1905; Wohlgemut et al. 1970; Hazen et al. 1978; Agger et al. 1985). |
| *Aeromonas* sp. * | Proteobacteria | Aeromonadaceae | 25-50  100-150  150-200  450-1000 | 12 | 100 | 100  98.53 | / |
| *Aeromonas veronii* | Proteobacteria | Aeromonadaceae | 100-150 | 1 | 100 | 100 | Human pathogen; previously detected in aquatic ecosystems (Joseph et al. 1991). |
| *Arthrobacter* sp. | Actinobacteria | Micrococcaceae | 100-150  150-200 | 2 | 100 | 100  93.83 | / |
| *Arthrobacter woluwensis* | Actinobacteria | Micrococcaceae | 100-150  150-200  350-450 | 4 | 100 | 100  99.49 | Human pathogen (Bernasconi et al. 2004; Li et al. 2021) |
| *Chromobacterium violaceum* | Proteobacteria | Neisseriaceae | 250-350 | 1 | 100 | 91.25 | Human pathogen; previously detected in aquatic habitats (Ponte and Jenkis 1992; Yang and Li 2011). |
| *Chryseobacterium* sp. * | Bacteroidetes | Flavobacteriaceae | 100-150 | 3 | 100 | 100  98.99 | / |
| *Chryseobacterium viscerum* | Bacteroidetes | Flavobacteriaceae | 100-150 | 1 | 100 | 100 | Previously detected in rainbow trout (Zamora et al. 2012). |
| *Citrobacter* sp. | Proteobacteria | Enterobacteriaceae | 100-150 | 1 | 100 | 100 | / |
| *Cloacibacterium* sp. or *Chryseobacterium* sp. | Bacteroidetes | Flavobacteriaceae | 450-1000 | 1 | 100 | 98.45 | / |
| *Cloacibacterium* sp. or *Chryseobacterium* sp. | Bacteroidetes | Flavobacteriaceae | 450-1000 | 1 | 100 | 98.45 | / |
| *Delftia* sp. * | Proteobacteria | Comamonadaceae | 100-150 | 1 | 100 | 100 | / |
| *Eleftheria terrae* | Proteobacteria | Burkholderiaceae | 25-50 | 1 | 100 | 96.88 | Previously detected in soil (Ling et al. 2015). |
| *Elizabethkingia anopheles* * | Bacteroidetes | Weeksellaceae | 200-250 | 1 | 100 | 100 | Human pathogen; previously detected in adult *An. gambiae* (Kämpfer et al. 2011; Lau et al. 2016). |
| *Escherichia coli* * | Proteobacteria | Enterobacteriaceae | 50-100  100-150  150-200 | 8 | 100 | 100 | Intestinal bacteria (Kaper et al. 2004); previously detected in adult *An. funestus* (Straif et al. 1989). |
| *Flavobacterium* sp. * | Bacteroidetes | Flavobacteriaceae | 50-100 | 1 | 100 | 100 | / |
| Fluviicola kyonggii | Bacteroidetes | Flavobacteriaceae | 50-100 | 1 | 100 | 100 | Previously detected in forest soil (Dahal and Kim 2018). |
| *Herbaspirillum* sp. | Proteobacteria | Oxalobacteraceae | 150-200 | 1 | 100 | 100 | / |
| *Hydrogenophaga pseudoflava* | Proteobacteria | Comamonadaceae | 150-200 | 2 | 100 | 100 | Previously detected in the mid gut of adult *An. gambiae* (Straif et al. 1989). |
| *Hydrogenophaga* sp. * | Proteobacteria | Comamonadaceae | 100-150 | 1 | 100 | 99.13 | / |
| *Kangiella koreensis* | Proteobacteria | Kangiellaceae | 450-1000 | 1 | 100 | 94.16 | Previously detected on a tidal flat (Yoon et al. 2004). |
| *Klebsiella oxytoca* | Proteobacteria | Enterobacteriaceae | 350-450 | 1 | 100 | 99.60 | Human pathogen (Reiss et al. 2000); previously detected in *Aspidimorpha miliaris* (Shil et al. 2014). |
| *Klebsiella* sp. | Proteobacteria | Enterobacteriaceae | 50-100 | 1 | 100 | 100 | / |
| *Kocuris rhizophila* | Actinobacteria | Micrococcaceae | 100-150 | 1 | 100 | 85.43 | Human pathogen (Moissenet et al. 2012) and fish pathogen (Pękala et al. 2018). |
| *Kocuria rosea* | Actinobacteria | Micrococcaceae | 100-150 | 1 | 100 | 99.02 | Human pathogen (Moreira et al. 2015). |
| *Limnobacter* sp. * | Proteobacteria | Burkholderiaceae | 150-200 | 2 | 100 | 99.36 | / |
| *Melaminivora* sp. or *Comamonas terrigena* | Proteobacteria | Comamonadaceae | 200-250 | 1 | 100 | 98.81  98.10 | / |
| *Microbakterium* sp. * | Actinobacteria | Mycobacteriaceae | 250-350 | 1 | 100 | 98.87 | / |
| *Micrococcus luteus* | Actinobacteria | Micrococcaceae | 100-150 | 2 | 100 | 100 | Human pathogen (Fosse et al. 1985). |
| *Nevskia lacus* or *ramos* | Proteobacteria | Xanthomonadaceae | 150-200 | 1 | 100 | 98.96 | / |
| *Nevskia ramosa* | Proteobacteria | Xanthomonadaceae | 50-100 | 1 | 100 | 100 | Previously detected in surface water (Pladdies et al. 2004). |
| *Nevskia* sp. | Proteobacteria | Xanthomonadaceae | 50-100  100-150 | 2 | 100 | 100 | / |
| *Pantoea* sp. | Proteobacteria | Erwiniaceae | 100-150  350-450  450-1000 | 4 | 100 | 100  98.44  96.63 | / |
| *Pantoea* sp. or *Enterobacter* sp. | Proteobacteria | Erwiniaceae or Enterobacteriaceae | 350-450 | 1 | 100 | 99.78 | / |
| *Pantoea* sp. or *Serratia sp.* | Proteobacteria | Erwiniaceae or Enterobacteriaceae | 450-1000 | 1 | 100 | 98.72 | / |
| *Paracoccus yeei* | Proteobacteria | Rhodobacteraceae | 100-150 150-200 | 2 | 100 | 100  99.4 | Human pathogen; previously detected in the salivary glands of adult *Ae. aegypti* (Arias and Clark 2019; Balaji et al. 2021). |
| *Paraoceanicella profunda* | Proteobacteria | Rhodobacteraceae | 150-200 | 1 | 100 | 97.04 | Previously detected in deep seawater (Liu et al. 2020). |
| *Paucibacter* sp. * | Proteobacteria | Unclassified Paucibacter | 100-150 | 1 | 100 | 99.17 | / |
| *Pelomonas* sp. or *Paucibacter* sp. | Proteobacteria | Comamonadaceae or Unclassified Paucibacter | 50-100 | 1 | 100 | 100 | / |
| *Perlucidibaca* sp. | Proteobacteria | Moraxellaceae | 350-450  450-1000 | 2 | 100 | 100  98.97 | / |
| *Pseudomonas anguilliseptica* | Proteobacteria | Pseudomonadaceae | 50-100 | 1 | 100 | 100 | Fish pathogen (Wiklund and Bylund 1990). |
| *Pseudomonas luteola* | Proteobacteria | Pseudomonadaceae | 100-150  150-200 | 2 | 100 | 100  99.49 | Human pathogen; previously detected in humid environments (Kostmann et al. 1990; Altinok et al. 2007). |
| *Pseudomonas* sp. * | Proteobacteria | Pseudomonadaceae | 100-150 | 1 | 100 | 100 | / |
| *Pseudomonas stutzeri* or *luteola* | Proteobacteria | Pseudomonadaceae | 150-200 | 1 | 100 | 99.37 | / |
| *Ralstonia* sp. | Proteobacteria | Burkholderiaceae | 150-200 | 1 | 100 | 98.97 | / |
| *Roseomonas mucosa* | Proteobacteria | Acetobacteraceae | 50-100 | 1 | 100 | 100 | Human pathogen; previously detected on human skin (Romano-Bertrand et al. 2016). |
| *Serratia* sp. or *Yersinia* sp. | Proteobacteria | Enterobacteriaceae or  Yersiniaceae | 150-200 | 1 | 100 | 100 | / |
| Uncultured alpha proteobacterium | Proteobacteria | — | 1000-2200 | 1 | 91 | 91.44 | / |
| Uncultured bacterium * | — | — | 50-100  150-200 | 8 | 100 | 100  98.78  97.44 | / |
| Uncultured *Shewanella* | Proteobacteria | Shewanellaceae | 25-50 | 1 | 100 | 100 | / |
| *Vogesella* sp. * | Proteobacteria | Chromobacteriaceae | 150-200 | 1 | 100 | 100 | / |
| *Wolbachia pipientis* * | Proteobacteria | Anaplasmataceae | 25-50  50-100  100-150  150-200  450-1000  1000-2200 | 18 | 100 | 100  98.57 | Previously detected in adult *Ae. albopictus* (Wiwatanaratanabutr 2013). |
| *Wolbachia* sp. * | Proteobacteria | Anaplasmataceae | 450-1000 | 2 | 100 | 99.63 | / |
| *Yersinia massiliensis* or *frederiksenii* | Proteobacteria | Yersiniaceae | 450-1000 | 2 | 100 | 99.85 | / |
| *Yersinia* sp. | Proteobacteria | Yersiniaceae | 150-200  200-250  450-1000 | 5 | 100 | 100  99.54 | / |
| Eukaryotes | | | | | | | |
| *Besnoitia besnoiti* or *Toxoplasma gondii* | Apicomplexa | Sarcocystidae | 200-250 | 1 | 100 | 82.03  81.57 | / |
| *Candida sake* * | Ascomycota | incertea sedis | 100-150 | 2 | 100 | 100 | Previously detected in oral cavity of HIV-positive people (Hoegl et al. 1998). |
| *Candida* sp. | Ascomycota | incertea sedis | 50-100  100-150 | 3 | 100 | 100 | / |
| *Cladosporium* sp. | Dothideomycetes | Mycosphaerellaceae | 150-200 | 1 | 100 | 100 | / |
| *Conidiobolus coronatus* | Zoopagomycota | Ancylistaceae | 150-200 | 1 | 100 | 100 | Human pathogen; previously detected on dead leaf (Fischer et al. 2008). |
| *Dinobryon* sp. * | Stramenopiles | Dinobryonaceae | 50-100 | 1 | 100 | 100 | / |
| *Entomophthora muscae* | Zoopagomycota | Entomophthoraceae | 150-200 | 1 | 100 | 99.46 | Insect pathogen; affects, e.g., houseflies (Watson et al. 1993; Becher et al. 2018). |
| Fungus | — | — | 50-100 | 1 | 100 | 100 | / |
| *Giradia tigrina* | Plathelminthes | Planariidae | 25-50 | 1 | 100 | 100 | Previously detected in freshwater (Oliveira et al. 2018). |
| *Hemimarginula pumila* | Mollusca | Fissurellidae | 25-50 | 1 | 100 | 95.45 | Marine mollusc (Nogueira et al. 2021). |
| *Opisthonecta* sp. | Peritrichia | Opisthonectidae | 250-350 | 1 | 100 | 100 | / |
| *Porites lichen* | Cnidaria | Poritidae | 100-150 | 1 | 100 | 96.15 | Hard coral. |
| *Rhizopus* sp. | Mucoromycota | Mucoraceae | 50-100 | 1 | 100 | 100 | / |
| *Syphacia* sp. | Nematoda | Oxyuridae | 50-100 | 1 | 100 | 100 | / |
| Uncultured fungus * | — | — | 50-100 | 3 | 100 | 100 | / |
